# Glucose sensing and homeostasis by adipocyte GPCR

**DOI:** 10.3389/fendo.2025.1657747

**Published:** 2025-09-18

**Authors:** Nazmul Hasan, Kavaljit H. Chhabra

**Affiliations:** ^1^ Department of Pharmacology and Nutritional Sciences, University of Kentucky, Lexington, KY, United States; ^2^ Barnstable Brown Diabetes and Obesity Research Center, University of Kentucky, Lexington, KY, United States

**Keywords:** glucose sensing, glucose homeostasis, adipose tissue, GPCR (G protein coupled receptor), obesity, diabetes, metabolism, glucose transport

## Abstract

The adipose tissue regulates energy homeostasis, which is one of the vital processes for organismal survival, and its dysregulation causes metabolic diseases including obesity and type 2 diabetes. Glucose is utilized by the adipose tissue for energy production and storage to regulate systemic glucose homeostasis. The G-protein-coupled receptors (GPCRs) expressed in the adipose tissues play a crucial role in adipocyte function by responding to hormonal, neural, and metabolic signals; thereby, influencing insulin sensitivity, glucose uptake and lipid metabolism. The specific contribution of adipocyte GPCRs to glucose sensing and its utilization is incompletely understood. Therefore, in this review we explore the diverse molecular and integrative mechanisms through which GPCR signaling in the adipose tissue senses glucose to regulate systemic glucose homeostasis. We first discuss the major GPCR families that modulate intracellular second messenger cascades in response to glucose and nutrient availability in the adipose tissue, and their metabolic implications in pathophysiological conditions like obesity and diabetes. These GPCRs regulate glucose sensing, lipid metabolism, adipokine secretion, and thereby coordinating metabolic responses with other central and peripheral tissues including the brain, pancreas, intestine and liver. Subsequently, we review the molecular mechanisms through which the adipocyte GPCR regulates systemic glucose homeostasis, from glucose sensing to its utilization. Determining how the GPCRs in the adipose tissue sense glucose will offer new and better therapeutic approaches for treating metabolic diseases including diabetes and obesity.

## Introduction

G-protein-coupled receptors (GPCRs) are the largest known cell surface receptor family in humans, which transmit extracellular signals (such as presence or absence of hormones, metabolites, neurotransmitters, sensory stimuli such as taste and odors) to inside of the cell to regulate physiological processes. Structurally, they are composed of seven transmembrane α-helical domains in addition to an extracellular amino- and an intracellular carboxy-terminus, therefore they are also known as seven-transmembrane receptors (1). This diverse group of receptors responds to their specific ligands and thus exerts their physiological functions. Upon ligand binding, they undergo some conformational changes to activate intracellular G-proteins (i.e., G_s_, G_i_, G_q/11_, or G_12/13_). These changes include the dissociation of Gα from the receptor and the Gβγ dimer, and the subsequent exchange of GTP for the bound GDP, which leads to Gα activation. The activated Gα then influences various downstream intracellular signaling and ultimately affects cellular function (2). At least 800 GPCRs have been identified in humans (1, 3), which are generally classified into five different groups according to the GRAFS (Glutamate, Rhodopsin, Adhesion, Frizzled/Taste2 and Secretin) classification system (4). These receptors are involved in nearly every physiological process, including the metabolic pathways, which makes them attractive targets for drug development. Over one-third of the drugs approved by the U.S. Food and Drug Administration target GPCRs to treat a variety of disorders (5).

Glucose is a vital macronutrient for organismal survival, providing fuel for energy production and carbon skeletons for various cellular components. To effectively sense and respond to changes in circulating glucose levels, the body employs a combination of hormonal signaling, neuronal pathways, and cellular mechanisms, which are essential for regulating glucose and energy homeostasis. Cellular glucose utilization is facilitated by the glucose sensors and receptors, both centrally and peripherally. Centrally, the brain hypothalamus and brainstem regions are well-known sites for glucose sensing and uptake, primarily through the glucose-excited (GE) and glucose-inhibited neurons (6–9). Peripherally, critical sites for glucose homeostasis include the pancreas, liver, skeletal muscle, kidneys, and the adipose tissue (10). A number of GPCRs have been identified as regulators of glucose homeostasis. For example, glucagon-like peptide-1 receptor (GLP-1R) is a widely studied GPCR, activated by GLP-1 in response to nutrients (e.g., glucose), which increase pancreatic insulin secretion and lowers blood glucose levels (11–13). Taste 1 receptors, particularly TAS1R2 and TAS1R3, another GPCR family members, are also involved in glucose sensing in pancreas, intestine, and skeletal muscle, and control glucose metabolism (14–16). Adhesion G-protein-coupled receptor L1 (ADGRL1) was recently reported to function as a hypothalamic glucose receptor that controls energy homeostasis in mice (17). ADGRL1-deficiency increases food intake, impairs glucose sensing and homeostasis, and causes obesity in mice (17, 18). These findings were also confirmed in pathogenesis of human obesity (18). Glucose-ADGRL1 binding was also validated using different methods (19) and the differences between available transgenic mouse models targeting Adgrl1 were discussed recently (20).

Although the contribution of GPCRs to glucose homeostasis is widely recognized, the precise role of adipocyte GPCR signaling in direct glucose sensing is incompletely understood. In this review, we provide an overview of the current understanding of GPCRs in the adipose tissue biology with a focus on their role in glucose sensing and homeostasis. First, we summarize the key GPCRs expressed in various adipose tissue depots and their metabolic implications in the pathogenesis of obesity and diabetes. Subsequently, we discuss the signaling mechanisms through which the adipocyte GPCRs sense glucose, respond to different glucose levels, and interact with other tissues to regulate overall energy balance.

## Adipose tissue types and their association with metabolic diseases

White adipose tissue (WAT), brown adipose tissue (BAT), and beige or brite (brown-in-white) adipose tissue are the three major adipose tissue types in mammals, with distinct morphological and functional characteristics. WAT comprises the highest portion of body fat and be further subdivided into subcutaneous and visceral WAT, according to their anatomical location (21, 22). Subcutaneous WAT is found under the skin, while visceral WAT resides in the abdominal cavity and surroundings of different intrabdominal organs. WAT acts as an energy storage depot, as it stores excess energy as triglycerides (TAGs), which are released as free fatty acids and glycerol during energy deficits. Subcutaneous WAT preferably stores excess fats and expands in size by hypertrophy and/or hyperplasia. But when this expansion halts due to reaching the limit or impairment of the expandability, fats start to deposit in visceral depots and other organs like kidneys, liver, heart, skeletal muscle, and pancreas. This ectopic fat deposition in non-fat tissues exacerbates lipotoxicity, resulting in insulin resistance, localized and systemic inflammation, and apoptotic cell death (23). Development of metabolic syndromes, including insulin resistance due to this excess visceral fat deposition is well known (24, 25) and leads to cardiovascular diseases and type 2 diabetes (26–28). Moreover, dysregulation in WAT’s endocrine functions also contributes to these metabolic diseases (28, 29).

BAT, the second adipose tissue type, was historically considered to only exist in hibernating animals, rodents, and to a lesser extent in infants, but its presence in adult humans has now been confirmed (30–33). Its amount is comparatively smaller (<3% of total fat mass) than the WAT and located in cervical, supraclavicular, axillary, mediastinal, paraspinal, and abdominal areas (32, 33). BAT is a metabolically highly active tissue that dissipates excess energy, mostly by thermogenesis involving its unique uncoupling protein 1 (UCP1). In addition to the well-established thermogenic property of BAT, UCP1-independent thermogenesis has also been reported recently (34–36). Like BAT, beige/brite adipose tissue exerts similar roles in thermogenesis and energy metabolism, mostly found in the subcutaneous WAT depots of rodents, and in cervical and supraclavicular regions in adult humans (37, 38). Beyond chronic cold exposure, other factors like adrenergic stimulation, diet, and exercise can also activate their thermogenic programming (34, 39, 40). These adipose tissues utilize glucose and fatty acids as fuel sources and play crucial roles in metabolic homeostasis (34, 41). Their activation improves insulin sensitivity and glucose uptake, increases lipolysis and fatty acid beta-oxidation, and reduces ectopic fat deposition and systemic inflammation (31, 34, 42–44). In contrast, dysfunction or inadequate activation of these adipose tissues reduces energy expenditure, and therefore, exacerbating metabolic and obesity-related complications. For example, BAT whitening - a condition when BAT loses its morphological and molecular characteristics and acts like WAT- exacerbates obesity complications in mice (45).

## Major GPCRs in the adipose tissue and their roles in glucose homeostasis

About 250 GPCRs have been identified in human (subcutaneous) WAT, while mice express over 270 and 290 GPCRs in WAT and BAT, respectively (46). Here, we are focusing on the major GPCRs, summarized in **Table 1**, involved in adipose tissue regulation of glucose and energy homeostasis.

**Table 1:** Major adipocyte GPCRs involved in regulating glucose homeostasis

GPCR	Endogenous ligand(s)	Signating molecules	Function	References
White adipose tissue (WAT)				
Alpha-adrenergic receptors				
α1-AR	Epinephrine nor-epinephrine	Gq PI3K/PKC	Stimulates glucose uptake and lactate production	(47–50)
Beta-adrenergic receptors				
β3-AR	Epinephrine nor-epinephrine	Gs, cAMP/PKA Gi, cAMP/PKA/MAPK	Stimulates glucose uptake, lipolysis, WAT browning	(51–54)
Dopamine receptors				
D2-like	Dopamine	Gi, cAMP/PKA	Enhances leptin production Stimulates glucose uptake	(55–57)
Adenosine receptors				
A1	Adenosine	Gi, cAMP/PKA	Inhibits lipolysis; regulates insulin sensitivity and glucose uptake; increases leptin secretion	(58, 59, 60–62)
A2B	Adenosine	Gs, cAMP/PKA	Prevents adipose tissue inflammation and insuin resistance	(63)
Free fatty acid receptors				
FFAR4 (GPR120)	Medium- and long- chain fatty acids	Gq, PI3K/Akt	Promotes insulin sensitization and enhances glucose uptake, WAT browning	(64–66)
Brown adipose tissue (BAT)				
Alpha-adrenergic receptors				
α1-AR	Epinephrine nor-epinephrine	Gq, PI3K/PKC	Promotes glucose uptake and thermogenesis	(50)
Beta-adrenergic receptors				
β1-AR	Epinephrine nor-epinephrine	Gs, cAMP/PKA/PI3K	Adipocytes differentiation, glucose uptake and thermogenesis	(50, 67, 68)
β2-AR	Epinephrine nor-epinephrine	Gs, cAMP/PKA	Enhances glucose uptake and BAT activity	(69–71)
β3-AR	Epinephrine nor-epinephrine	Gs, Gi, cAMP/PKA	Enhances insulin sensitivity, glucose uptake and thermogenesis	(72–75)
Adenosine receptors				
A2A A2B	Adenosine	Gs, cAMP/PKA	Enhances BAT activity and energy expenditure	(76, 77)

Beta-adrenergic receptors (β-ARs: β1, β2, and β3 subtypes) are one of the highly expressed and well-investigated GPCRs in both human and mouse adipose tissues (68). Ligand (e.g., norepinephrine) mediated activation of β-ARs, particularly β3-AR, promotes lipolysis, mitochondrial respiration, and browning of WAT (51, 54, 78, 79). β3-AR activation also stimulates glucose uptake in WAT through insulin-dependent and -independent mechanisms (80). Pharmacological stimulation with the β3-AR agonist CL316,243 increases glucose uptake in rats mesenteric WAT (53), while Trecadrine (another β3-AR agonist) promotes insulin-dependent glucose uptake in cultured rat white adipocytes (81). Conversely, in brown and beige adipose tissue, β3-AR activation enhances thermogenesis by upregulating uncoupling protein 1 (UCP1) expression, and increasing lipolysis-derived free fatty acids and glucose utilization (72, 82, 83). These free fatty acids and glucose serve as fuels for the adaptive thermogenic process, which is crucial for maintaining the energy balance. However, β3-AR-mediated glucose uptake in BAT primarily takes place through dual mechanisms: cAMP-mediated upregulation of GLUT1 expression and mTORC2-dependent translocation of GLUT1 to the plasma membrane, independent of the classical insulin/PI3K/Akt pathway (84–86). Interestingly, β3-AR–stimulated glucose uptake occurs even in the absence of UCP1 (87), indicating that acute glucose uptake is not strictly coupled to thermogenesis but rather mediated by distinct signaling mechanisms. Clinical trials have also confirmed that treatment with mirabegron, a β3-AR agonist, helps to improve metabolic health by enhancing insulin sensitivity, WAT lipolysis, and BAT thermogenesis (72–74). While the glucoregulatory role of β2-AR in WAT remains unclear, it is well characterized in BAT. β2-AR stimulation by its selective agonist, salbutamol, increases both glucose uptake and BAT activity in mice (69), which is further confirmed in human BAT as well (70, 88). In contrast, β1-AR signaling is primarily linked to WAT lipolysis (89) and adipocyte differentiation in both WAT and BAT (67, 68). Although β1-AR may not be the most significant AR in terms of maintaining glucose homeostasis, it has been shown to facilitate glucose uptake in cultured brown adipocytes lacking β3-AR (50). Notably, the distribution and function of β-AR subtypes differs between species, for example, in human, β1- and β2-ARs, but not β3-AR (90), mediate lipolysis in WAT (71). On the other hands, all three subtypes of β-ARs are found in both WAT and BAT of rodents.

In addition to β-ARs, α-adrenergic receptors (α1- and α2-AR) are also expressed in both white and brown adipocytes. Stimulation of α1-AR increases glucose uptake and lactate production in rat white adipocytes that were resistant to insulin, indicating an insulin independent glucose uptake mechanism (47, 48). In support of this finding, α1-AR stimulated increase in glucose uptake and metabolism were also confirmed in human WAT (49, 91). This process is thought to be mediated via the phosphoinositide 3-kinase (PI3K)/protein kinase C (PKC) pathway, which is activated through α1-AR signaling induced by specific agonists and/or neurotransmitters (92). However, while β-adrenoceptors are often considered the primary regulators of thermogenesis in BAT, α-adrenoceptors and their downstream signaling pathways are also crucial for this process. A study conducted by Chernogubova et al. showed that stimulation of α1-adrenergic receptor, in association with β1-AR stimulation, is also involved in glucose uptake using the PI3K/PKC signaling in cultured β3-KO brown adipocytes, and can compensates the lack of β3-AR signaling (50). In addition, dopamine receptors (D1- and D2-subtypes) are also expressed in both humans and rodents white and brown adipocytes, which are involved in the regulation of glucose uptake and lipid metabolism, and adipocyte browning as well (56, 57, 93–95).

Both human and rodent adipose tissue express adenosine receptors (A1, A2A, A2B, and A3 subtypes), which bind to different G-proteins in adipocytes to stimulate or inhibit adenylyl cyclase activity and, consequently, influence glucose homeostasis. The A1-adenosine receptors are highly expressed in WAT and have inhibitory effects on lipolysis and may promote fat storage in adipocytes (96–98). Although a number of studies have investigated the role of A1-adenosine signaling in insulin action and glucose metabolism in white adipocytes *in vitro*, the findings remain controversial. Pharmacological activation of A1 adenosine receptor in white adipocytes isolated from rats showed decreased insulin sensitivity and glucose uptake (92, 99). Conversely, others found that adenosine increases insulin-stimulated glucose uptake and lipogenesis (62, 100, 101). *In vivo* studies in rodents have suggested that A1-adenosine receptor activation in WAT improves glucose tolerance and insulin sensitivity, and its deficiency leads to glucose intolerance and impaired insulin action (60, 62, 102). Although A2A and A2B adenosine receptors are expressed in both WAT and BAT; A2A is predominantly expressed in BAT and facilitates thermogenesis and promotes energy expenditure. Deletion or inhibition of A2A receptors reduces BAT thermogenesis, while their activation promotes WAT browning in mice (76). A2B receptors are also abundant in BAT and regulate adipogenesis and BAT functioning in mice and humans (77, 97). The activation/stimulation of A2B receptors protects mice from high-fat diet-induced obesity by increasing BAT-mediated energy expenditure (77). Moreover, it also prevents insulin resistance by inhibiting inflammation in the adipose tissue and regulates glucose homeostasis in diabetic and obese conditions (63).

Free fatty acid receptors (FFARs) are another group of GPCRs highly expressed in the adipose tissue and are crucial for regulating glucose homeostasis by influencing insulin sensitivity. These receptors include FFAR1 (GPR40), FFAR2 (GPR43), FFAR3 (GPR41), and FFAR4 (GPR120). Each receptor is activated by different types of fatty acids, with FFAR1 and FFAR4 responding to medium- and long-chain fatty acids, while FFAR2 and FFAR3 are primarily activated by short-chain fatty acids (SCFAs), and leading to several metabolic outcomes (103, 104). For instance, GPR120 (FFAR4) activation enhances insulin sensitivity and promotes anti-inflammatory responses in adipocytes, which is particularly important in the context of obesity and insulin resistance (65, 66). Dysfunction of GPR120 has been linked to obesity and metabolic disorders, as evidenced by studies demonstrating that its ablation leads to increased adiposity and insulin resistance in both mice and humans (64, 65, 105). Moreover, both increased BAT activity and WAT browning were also reported in mice due to GPR120 activation, which supports its role in thermogenesis (106). FFAR2 and FFAR3 activation have also been shown to influence lipolysis and energy expenditure in the adipose tissue. For example, acetate (a short-chain fatty acid) mediated activation of FFAR2 inhibits lipolysis in human white adipocytes by reducing phosphorylation of hormone-sensitive lipase (107). Furthermore, FFARs can regulate the secretion of adipokines, which are critical for maintaining metabolic balance and responding to changes in nutrient availability (65, 66).

Expression of adhesion GPCRs (aGPCRs) is also evident (about 37% of all aGPCRs) in human and mouse adipose tissues, where a substantial proportion of these receptors is differentially regulated under conditions of obesity and high-fat diets (108). In addition to their expression patterns, the functional relevance of aGPCRs in the adipose tissue is becoming increasingly clear. These receptors are implicated in various signaling pathways that regulate adipocyte function, including adipogenesis, lipolysis, and inflammation (109, 110). For instance, GPR116 has been identified as a key player in mediating insulin-sensitizing effects in white adipose tissue (111). While RNA sequence data revealed the presence of several aGPCRs in the adipose tissue, including ADGRL1-3/LPHN1-3, CD97, GPR125, GPR56, GPR64, and GPR97, their functional relevance in adipose biology is yet to be investigated. Although the role of ADGRL1 in regulating glucose and energy homeostasis was reported recently (17, 18) the contribution of adipocytic ADGRL1 to glucose sensing and responding to changes in blood glucose levels remains to be determined. It is likely that the local adipocyte ADGRL1 is involved in glucose signaling pathways and pathogenesis of type 2 diabetes and obesity.

In summary, the extensive diversity of GPCRs expressed in the adipose tissue and their ability to mediate complex signaling pathways to regulate glucose homeostasis underscore their potential as therapeutic targets for type 2 diabetes, obesity and related metabolic disorders. Future research is expected to elucidate the specific roles of individual GPCRs in the adipose tissue function and their interactions with other metabolic pathways in regulating energy and glucose homeostasis.

## Glucose sensing by adipocyte GPCRs

**Figure 1:**
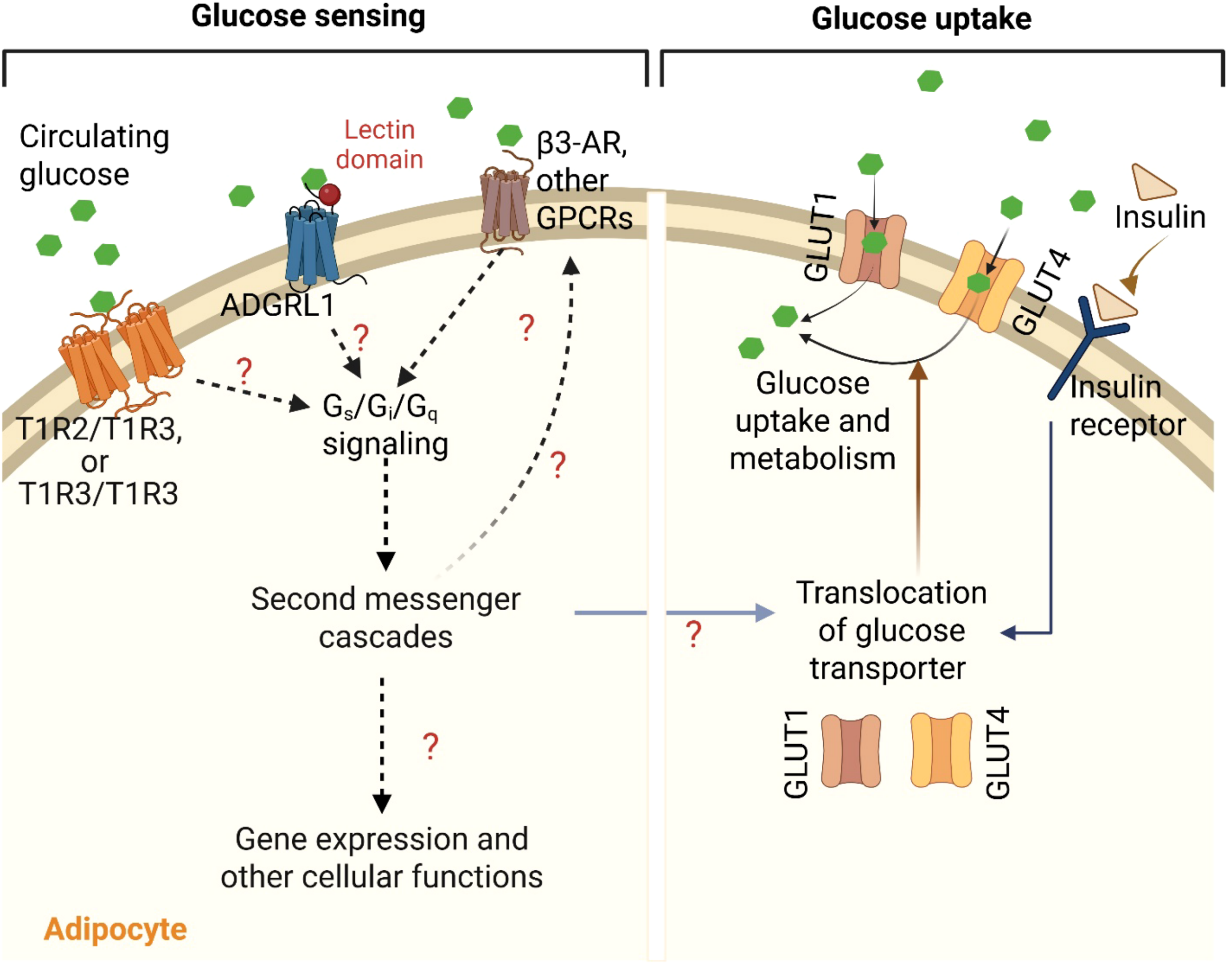
G-protein coupled receptors (GPCRs)-mediated sensing and uptake of glucose in adipocyte. Various GPCRs such as homo- or heterodimers of taste receptors (T1R2/T1R2 and/or T1R2/T1R3) and adhesion G-protein coupled receptor L1 (ADGRL1) sense and bind to the circulating blood glucose and activate Gs, Gi and/or Gq signaling pathways. The resulting second messenger cascades may then regulate insulin sensitivity, translocation of glucose transporters (GLUT1 and GLUT4), activities of other GPCRs including β3-adrenergic receptor, and other cellular and molecular functions in the adipocytes to control glucose uptake, either by insulin-dependent or -independent mechanisms. This figure was created with BioRender.com.

Although glucose sensing and uptake may seem to be the same phenomenon, they occur at different spatial and temporal levels (**Figure 1**) to complement each other or to accomplish their individual functions. For example, adipocyte glucose sensing involves monitoring systemic or local glucose levels by plasma membrane receptors including GPCRs to influence downstream signaling pathways and thereby, maintain glucose homeostasis. In contrast, glucose uptake and utilization facilitate intracellular metabolism and energy production to support cell growth and proliferation.

The adipose tissue utilizes a significant amount of glucose for either storage or energy production. The processes of glucose sensing and cellular glucose uptake are key steps involved in glucose homeostasis by the adipose tissue. In postprandial state, upon sensing blood glucose levels, pancreatic beta-cells secrete insulin that binds and activates insulin receptors on the adipocyte’s membrane. Activated insulin receptors then initiate a signaling cascade involving phosphoinositide 3-kinase (PI3K) and protein kinase B (Akt), which are critical for the translocation of GLUT4 on the cell surface and thus, allows glucose entry into the adipocytes from the blood (112, 113). While GLUT4 is the predominant glucose transporter in adipocytes, another transporter -GLUT1 - also contributes to an insulin-independent glucose uptake (114). However, beyond insulin signaling, GPCRs act as integral mediators of glucose sensing and metabolism within the adipose tissue, influencing glucose sensing, insulin sensitivity and glucose uptake through diverse signaling pathways. Several GPCR families in adipocytes participate in glucose sensing through Gq, Gi, and/or Gs signaling. Chemogenetic stimulation of Gs signaling in adipocytes resulted in a significant reduction in blood glucose levels, indicating its role in improving glucose tolerance (115). Likewise, Kimura et al. have also reported the involvement of Gq signaling on glucose uptake in the adipose tissue to improve glucose homeostasis in mice (116). In addition to Gs and Gq signaling, Gi signaling is also crucial for maintaining glucose homeostasis in adipocytes. Wang et al. have shown that Gi signaling is essential for preserving insulin sensitivity and regulating glucose metabolism in the adipose tissue (117). Therefore, the balance between these GPCR signaling pathways is crucial for the proper metabolic function of adipocytes.

How adipocytes detect changes in blood glucose levels, and how adipocytic GPCRs and their signaling pathways respond to these changes to regulate glucose homeostasis remain unclear. The sweet taste receptors (particularly T1R2 and T1R3) expressed in the adipose tissue are potential candidates for direct glucose sensing by adipocytes. Several studies have shown their roles in regulating glucose homeostasis, adipogenesis and lipolysis (118–121). Although the precise mechanism by which these taste receptors sense glucose in adipocytes is yet to be elucidated, their capacity to sense sugars including glucose by forming hetero (T1R2/T1R3)- and homo (T1R3/T1R3)-dimers in other tissues like intestine and skeletal muscle are well known (16, 122). Masubuchi et al. reported that activation of T1R3 homomeric receptors reduces insulin-induced GLUT4 translocation and glucose transport in a Gs-dependent, cAMP-independent manner (123).

T1R3 knockout mouse models exhibit impaired glucose clearance, reduced insulin sensitivity, and increased adiposity, highlighting the importance of T1R3 in maintaining glucose homeostasis (124, 125). Moreover, activation of T1R2/T1R3 can also indirectly impact glucose uptake by stimulating the release of incretin hormones like GLP-1, which enhances insulin secretion and glucose uptake in peripheral tissues, including the adipose tissue (126–128). Future studies targeting adipocyte-specific knockout of T1R2 and T1R3 may provide better mechanistic insights regarding how these GPCRs are involved in glucose homeostasis. In addition, ADGRL1 may also contribute to glucose sensing in adipocytes as the receptor was recently shown to bind and sense glucose in the hypothalamus (17, 20), which also warrants further investigation.

The involvement of β-ARs in regulating glucose uptake in adipocytes, either by insulin dependent or independent mechanisms, is well known as we discussed in the earlier section. For example, β3-AR, coupled to Gs protein, stimulates GLUT1 translocation to the membrane in brown adipocytes, increasing glucose uptake independently of insulin, through cAMP-dependent mechanisms and mTORC2 activation (84–86, 129). Similarly, ligand (salbutamol)-mediated activation of β2-AR, has also been shown to increase glucose uptake in human BAT, but not in WAT (70). In tissues such as the brain, liver, and intestine, the β-ARs are involved in glucose sensing (130–132), which may be tied to the role of the receptors in affecting glucose uptake by their interactions with glucose transporters through downstream signaling pathways. Based on these reports, we speculate that the effects of β-ARs on glucose uptake in the adipose tissue may be associated with glucose sensing via communications between the receptors and glucose transporters (**Figure 1**). This may explain the adaptability of the adipose tissue under different glucose levels.

Altogether, adipocyte GPCRs may contribute to direct glucose sensing in addition to their role in glucose uptake and metabolism in the adipose tissue (**Figure 1**). Determining the molecular mechanisms involved in interactions between adipocyte GPCRs, glucose sensing, glucose transporters, and glucose metabolites will help develop more effective strategies to manage metabolic disorders associated with impaired energy and glucose balance.

## GPCR-mediated crosstalk of the adipose tissue with other central and peripheral organs to regulate glucose homeostasis

### The brain-adipose tissue axis

Depending on the glycemic status, the hypothalamus regulates the secretion of the pancreatic hormones (e.g., insulin and glucagon) through the autonomic [parasympathetic (PNS) and sympathetic (SNS)] nervous systems to maintain euglycemia. The mechanisms governing PNS- and SNS-mediated insulin secretion have been comprehensively reviewed by Valentine S. Moullé (133). Neurotransmitters released due to the activation of these autonomic pathways, such as acetylcholine and adrenaline, activate specific GPCRs (e.g., muscarinic and adrenergic receptors) in pancreatic β-cells, triggering downstream signaling cascades through G-proteins (Gq, Gi, and Gs) to regulate insulin release. For instance, activation of α1-adrenergic receptor (α1-AR) and muscarinic receptor 3 (M3R) leads to Gq and Gi signaling, which enhances insulin secretion by increasing intracellular Ca²^+^ levels. Conversely, α2-adrenergic receptor (α2-AR)-mediated Gs signaling inhibits insulin secretion by elevating intracellular K^+^ levels (133). These pancreas-secreted hormones are then transported throughout the body via circulation and regulate systemic glucose homeostasis through different tissues including the adipose tissue.

**Figure 2:**
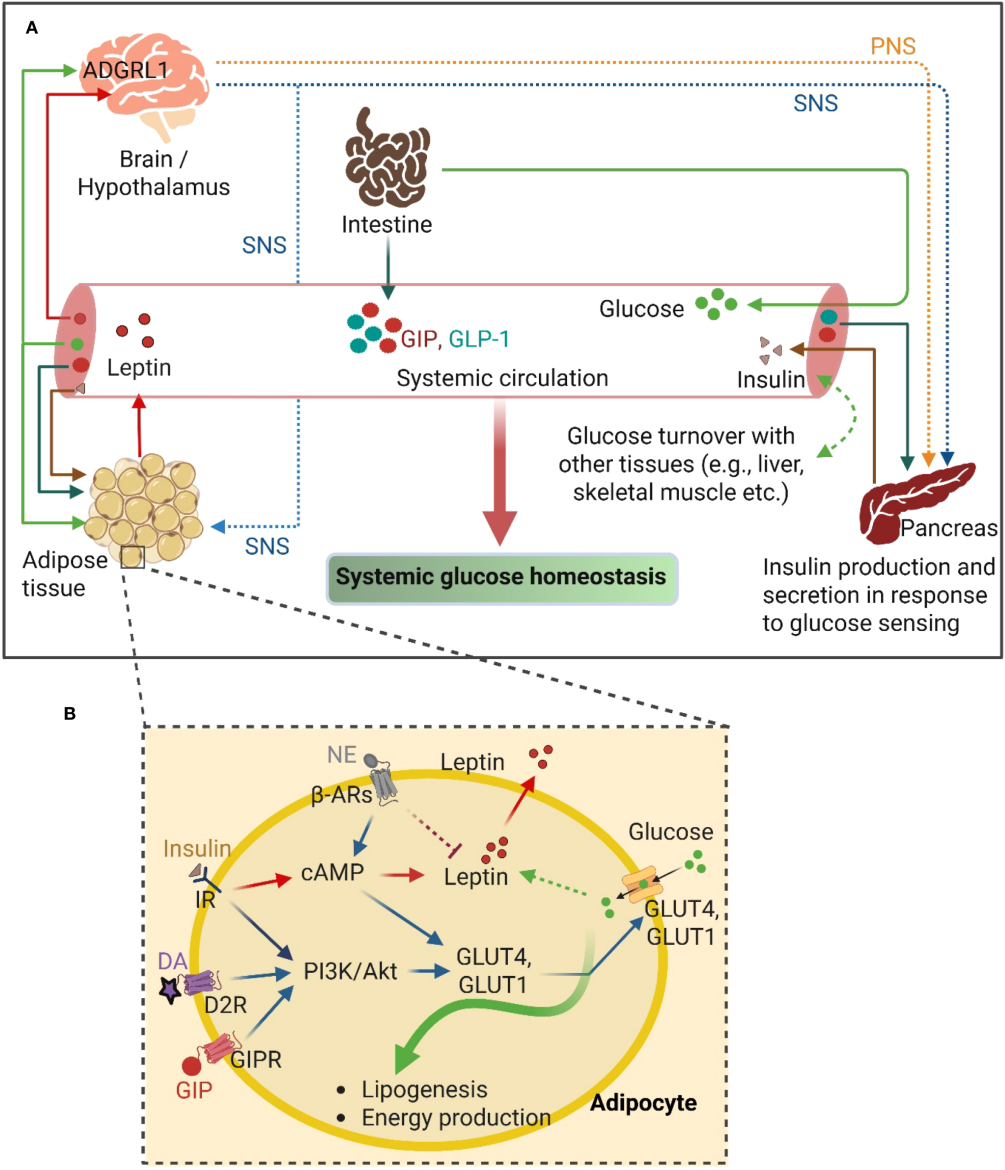
GPCR-mediated crosstalk between the adipose tissue and other organs to regulate glucose homeostasis. **(A)** The overview of the interconnecting pathways between adipose tissue and other central and peripheral organs, including the intestine and pancreas, regulating the systemic glucose homeostasis. **(B)** GPCR-mediated signaling mechanisms in adipocyte involving glucose uptake and utilization. PNS, Parasympathetic nervous system; SNS, Sympathetic nervous system; GIP, Glucose-dependent insulinotropic polypeptide; GIPR, Glucose-dependent insulinotropic polypeptide receptor; GLP-1, Glucagon-like peptide-1; NE, Norepinephrine; DA, Dopamine; D2R, Dopamine receptor D2; β-ARs, β-adrenergic receptors; PI3K, Phosphoinositide 3-Kinase; Akt, Protein kinase B; cAMP, Cyclic adenosine monophosphate; IR, Insulin receptor; GLUT4 and GLUT1, Glucose Transporter 4 and 1; respectively. ADGRL1, Adhesion G-protein coupled receptor L1. This figure was created with BioRender.com.

Given that glucose uptake in the adipose tissue is largely insulin-dependent (134) and the insulin sensitivity decreases with an increased adiposity (135, 136), an enhanced insulin secretion from the β-cells and/or an increase in insulin sensitivity are necessary for maintaining glucose homeostasis (137). This is accomplished through the combined contribution of the hypothalamus, β-cells, and the adipose tissue. For example, decreased insulin-dependent glucose uptake in WAT of hypothalamic ADGRL1-deficient mice was reported recently (17). The mice also had impaired insulin secretion probably associated with enhanced vagus nerve activity, since pancreatic vagotomy reversed insulin hypersecretion in the ADGRL1-deficient mice. Further studies are required to investigate whether hypothalamic ADGRL1 regulates SNS activity to influence insulin-induced glucose transport in adipocytes (**Figure 2**).

Norepinephrine secreted from the activated SNS functions through the adipocytic β3-AR to enhance glucose uptake and thermogenesis (87). In addition, dopamine, a neurotransmitter, secreted from SNS activation binds to the D2-like dopamine receptors (mainly D2R and D3R, members of the GPCR family) in the pancreatic beta-cells and negatively regulates glucose-stimulated insulin secretion (138–140). Dopamine also directly regulates glucose uptake in insulin-sensitive tissues, including WAT, liver, and skeletal muscle, acting through the dopamine receptors. Dopamine administration has been reported to directly enhance glucose uptake in WAT and the liver via D2R (57). D2R activation also modulates the secretion of adipokines, including leptin and adiponectin, the critical regulators of systemic energy balance (56) (**Figure 2**).

In addition to its roles in regulating insulin action and glucose uptake in adipose tissue, SNS also triggers others metabolic pathways including- lipolysis, browning of WAT and thermogenesis in adipose tissues, which are crucial for the maintenance of systemic glucose homeostasis. In WAT, SNS-mediated stimulation of adrenergic signaling promotes lipolysis and provides free fatty acids and glycerol for systemic energy supply and gluconeogenesis, respectively (78, 141, 142). Furthermore, adrenergic signaling also stimulates thermogenic UCP1 expression in brown and beige adipocytes, which facilitates thermogenesis (83). A significant amount of glucose and free fatty acids are utilized in this thermogenic process, and thereby improving systemic glucose clearance. Collectively, these SNS-driven processes integrate brain-adipose tissue communication to regulate lipid and glucose metabolism, ultimately contributing to whole-body energy homeostasis.

### The intestine-adipose tissue axis

Incretin hormones (GIP and GLP-1) are secreted from the intestinal cells upon glucose sensing by the gastrointestinal tract postprandially (143). The glucose homeostatic regulatory function of these incretins is largely mediated by their insulinotropic and glucagonotropic functions on the pancreas, such as by augmenting the insulin secretion from the pancreatic beta-cells (144, 145). At the pancreatic endocrine cells, GIP and GLP-1 bind to their respective G-protein coupled receptors (GIPR and GLP-1R), and transduce signal to increase cAMP production and protein kinase B (AKT) activation, which finally enhances insulin secretion from the beta-cells (146–148). Interestingly, the incretins - specifically GIP – also directly regulate glucose uptake in the adipose tissue by binding to GIPR in adipocytes. For instance, GIP-stimulated glucose uptake in 3T3-L1 adipocytes (in the presence of insulin) was reported over 20 years ago by Miyawaki et al (149). Later, Song et al. demonstrated that GIP has insulin-mimetic effects on glucose uptake in 3T3-L1 adipocytes, which is mediated through the activation of Akt via wortmannin (a potent inhibitor of PI3K)-sensitive pathway, at least partly, which promotes GLUT4 translocation to the adipocyte membrane to enhance glucose uptake (150). Although an earlier study reported that GIPR is predominantly expressed in non-adipocytes in the adipose tissue (151), recently Regmi et al. have reconfirmed the expression of functional GIPR in both human and mouse adipocytes (152). The authors demonstrated that activation of GIPR-signaling upon binding through GIP and/or tirzepatide (a dual agonist of GIPR/GLP-1R) enhances both insulin-dependent and -independent glucose uptake differentiated in human adipocytes (152). Moreover, abolishment of the gluco- and lipo-regulatory effects of GIP in lean human adipose tissue was demonstrated with the infusion of GIP(3-30)NH_2_, an antagonist of human GIPR, during hyperglycemic-hyperinsulinemic clamps (153). In contrast, BAT-specific deletion of GIPR in mice showed no significant alteration in glucose homeostasis (154). These findings indicate that GIPR signaling in BAT may be dispensable for glucose regulation, while GIPR signaling in WAT contributes to adipose tissue glucose uptake predominantly. Collectively, the adipose tissue receives glucose-sensing signals from the intestine through incretins to modulate the local adipocyte regulation of glucose homeostasis via GPCRs (**Figure 2**).

## Future perspective and concluding remarks

Most studies have focused on glucose uptake, utilization, and its metabolism to establish the role of the adipose tissue in regulating glucose homeostasis. Although great progress has been made in this area of research, glucose sensing aspects of the adipose tissue remain unclear. For example, the following questions are largely unaddressed: how does the adipose tissue sense blood or local glucose levels? What are the molecular mechanisms through which the adipose tissue responds to the changes in glucose levels to restore homeostasis? Investigating adipocytic GPCRs and associated transduction pathways including the transcription factors may provide novel insights into the molecular machinery involved in glucose sensing and responding to changes in systemic or local glucose levels. This topic will open new research avenues for investigating glucose signaling pathways in the adipose tissue independently of glucose metabolism or its transport.
